# Transitional Care Management: Evidence for Novel Implementation Models and Rehabilitation Implications

**DOI:** 10.1093/geroni/igaa057.2916

**Published:** 2020-12-16

**Authors:** Margaret Danilovich, Margaret Danilovich

## Abstract

The transition between healthcare settings is a complex process presenting challenges for effective and consistent communication between older adults, their caregivers, and healthcare providers. These challenges often result in adverse health events and re-hospitalizations. Further, once transitioned to home, older adults often need ongoing care management and support and evidence for models remains unclear as to the precise parameters of supports needed for comprehensive care. This symposium will provide an overview of the evidence for both interdisciplinary care management models and transitional care programs, present the implementation of a care management program for low income older adults at one social service agency, and provide evidence-based tools for older adult functional assessment and decision-making for transitional care. The speakers will present new tools from the American Physical Therapy Association home health toolbox that promote patient-centered health care decision-making to facilitate successful transitions that reduce resource use and hospital readmission. The speakers will also discuss the implementation of a care management program for older adults in a care gap (having too much income for Medicaid home and community-based services, but still <200% of the federal poverty line). An implementation framework for the needs assessment will be highlighted and 1-year program outcomes will be presented. Attendees will learn strategies for interprofessional collaboration, enhanced communication, and advocacy within the interprofessional team to facilitate improved care management and transitional services for older adults.

